# Supplementary material for: The Proteasomal Deubiquitinating Enzyme PSMD14 Regulates Macroautophagy by Controlling Golgi-to-ER Retrograde Transport
Source: Cells. 2020 Mar 23;9(3):777. doi: 10.3390/cells9030777 (PMC7140897; doi:10.3390/cells9030777)
Supplement: Supplementary file 1 [file cells-09-00777-s001.pdf]

Supplementary Materials

The proteasomal deubiquitinating enzyme PSMD14 regulates macroautophagy by controlling Golgi-to-ER retrograde transport

Bustamante HA., et al.

Figure S1. siRNA sequences directed against human PSMD14 used for Validation Stage.

Figure S2. Primer pairs sequences used for RT-qPCR.

Figure S3. The PSMD14 DUB inhibitor CZM increases the Golgi apparatus area.

Immunofluorescence microscopy analysis of the Golgi area in parental H4 cells treated for 4 h either with the vehicle (DMSO; Control) or CZM. The Golgi marker GM130 was used to determine the region of interest in each condition. Statistical significance was determined by Student's t-test. Bars represent the mean ± SEM (n =43 cells). ***P <0.001.

Figure S4. CZM causes the accumulation of KDELR1-GFP at the Golgi apparatus.

HeLa cells expressing KDELR1-GFP were either left untreated or treated with CZM for 30, 60 or 90 min. Cells were fixed and representative confocal images were acquired.

Figure S5. Effect of CZM on proteasome activity.

Parental H4 cells were treated either with the vehicle (DMSO; Control), CZM or MG132, for 90 min. Protein extracts were used to measure *in vitro* the Chymotrypsin-like peptidase activity of the proteasome. The enzymatic activity was quantified according to the cleavage of the fluorogenic substrate Suc-LLVY-AMC to AMC, and normalized to that of control cells. The statistical significance was determined by One-Way ANOVA, followed by Tukey's test. Bars represent the mean ± SD of biological replicates (n=3). **P <0.01; n.s., not significant.

Figure S6. Effect of CZM and MG132 on basal macroautophagy.

(A) Immunofluorescence microscopy analysis of the subcellular localization of LC3 in parental H4 cells treated with either with the vehicle (DMSO; Control), CZM for 4 h or MG132 for 6 h. Cells were fixed, permeabilized and stained with a rabbit polyclonal antibody to LC3B followed by Alexa-594-conjugated donkey anti-Rabbit IgG. Scale bar 10 μm. (B) Parental H4 cells were treated as in (A) and the protein extracts were analyzed by western blot with a polyclonal antibody to LC3B. Densitometric quantification of the protein levels of LC3B were depicted as the Ratio LC3B-II/LC3B-I. The statistical significance was determined by One-Way ANOVA, followed by Tukey's test. Bars represent the mean ± SD of biological replicates (n=3). ***P <0.001; n.s., not significant.

Figure S7. Distribution of RAB1A upon CZM treatment.

Immunofluorescence analysis of endogenous RAB1A in H4 parental cells treated either with vehicle (DMSO; Control) (A-C) or CZM for 4 h (D-F). Cells were fixed, permeabilized, and double stained with a rabbit

monoclonal antibody to RAB1A (clone D3X9S) (A and D) and a mouse monoclonal antibody to GM130 (clone35/GM130) (B and E), followed by Alexa-594-conjugated donkey anti-Rabbit IgG and Alexa-488-conjugated donkey anti-Mouse IgG. Merging of the images generated the third picture (C and F). Scale bar, 10 ◎m. (G) Quantitative analysis of the fraction of RAB1A colocalizing with GM130 under CZM treatment and compared to control cells. The statistical significance was determined by Student's t-test. Bars represent the mean ± SEM of the fluorescent signal per cell area (n=173 cells). *P< 0.05.

Figure S8. ATG9A is distributed in the swollen Golgi apparatus upon CZM treatment.

Immunofluorescence analysis of endogenous ATG9A in H4 parental cells treated either with the vehicle (DMSO; Control) (A-C) or CZM for 4 h (D-F). Cells were fixed, permeabilized, and double stained with a rabbit monoclonal antibody to ATG9A (clone EPR2450(2)) (A and D) and a mouse monoclonal antibody to GM130 (clone35/GM130) (B and E), followed by Alexa-594-conjugated donkey anti-Rabbit IgG and Alexa-488-conjugated donkey anti-Mouse IgG. Merging of the images generated the third picture (C and F). Scale bar, 10 ◎m. (G) Quantitative analysis of the fraction of ATG9A colocalizing with GM130 under CZM treatment and compared to control cells. The statistical significance was determined by Student's t-test. Bars represent the mean ± SEM of the fluorescent signal per cell area (n=93 cells). **P <0.01.

	Sequence
**siRNA #1**	(5´-GAACAAGUCUAUAUCUCUU-3´)
**siRNA #2**	(5´-GGCAUUAAUUCAUGGACUA-3´)
**siRNA #3**	(5´-AGAGUUGGAUGGAAGGUUU-3´)
**siRNA #4**	(5´-GAUGGUUGUUGGUUGGUAU-3´)

Figure S1

Target	Sequence
**hTBP1**	f(5´-TAGTCCAATGATGCCTTACG-3´)<br>r(5´-TGGTCAGAGTTTGAGAATGG-3´)
**hPSMD14**	f(5´-ACCTTAAGAGTTGTAGTTACTGACC-3´)<br>r(5´-TTTAACAGTGCCAGGGAAGAG-3´)
**hAPP**	f(5´-CCTAAAGCATTTCGAGCATG-3´)<br>r(5´-GTTTCCGTAACTGATCCTTG-3´)

Figure S2

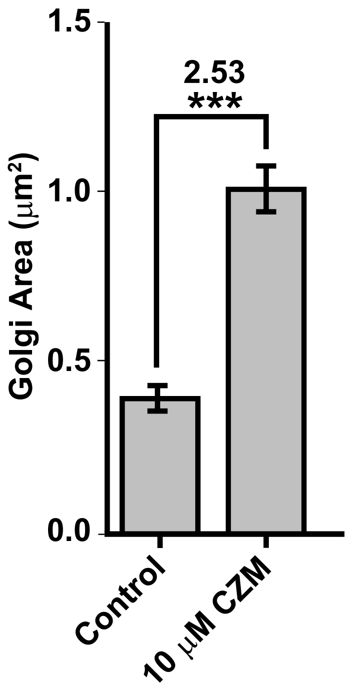

Figure S3

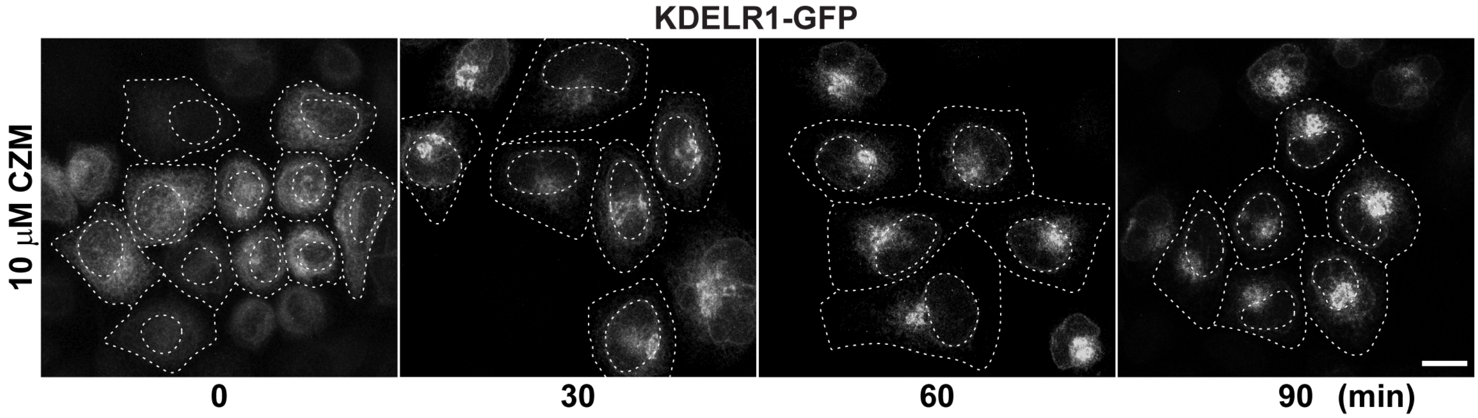

Figure S4

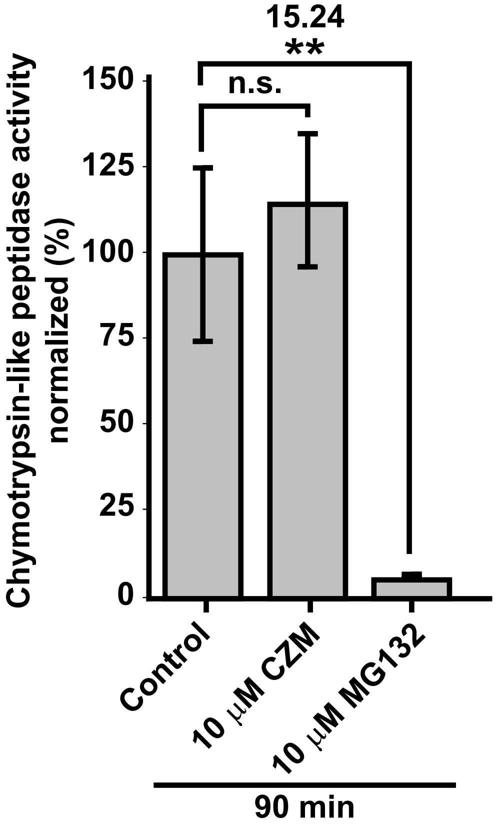
15.24
**
n.s.
Chymotrypsin-like peptidase activity normalized (%)
150
125
100
75
50
25
0
Control
10 μM CZM
10 μM MG132
90 min

Figure S5

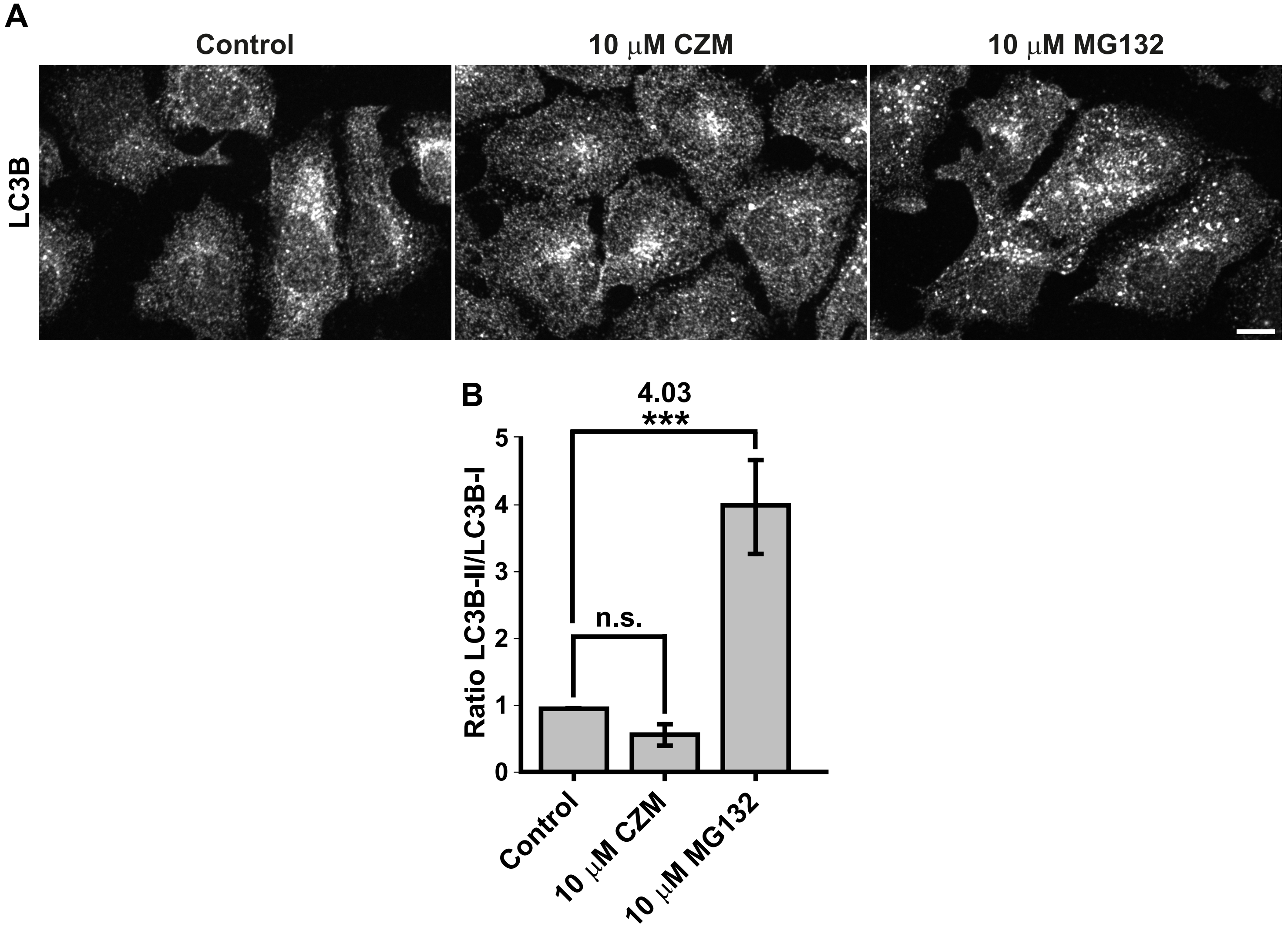
A
Control
10 μM CZM
10 μM MG132
LC3B
B
4.03
***
n.s.
Ratio LC3B-II/LC3B-I
5
4
3
2
1
0
Control
10 μM CZM
10 μM MG132

Figure S6

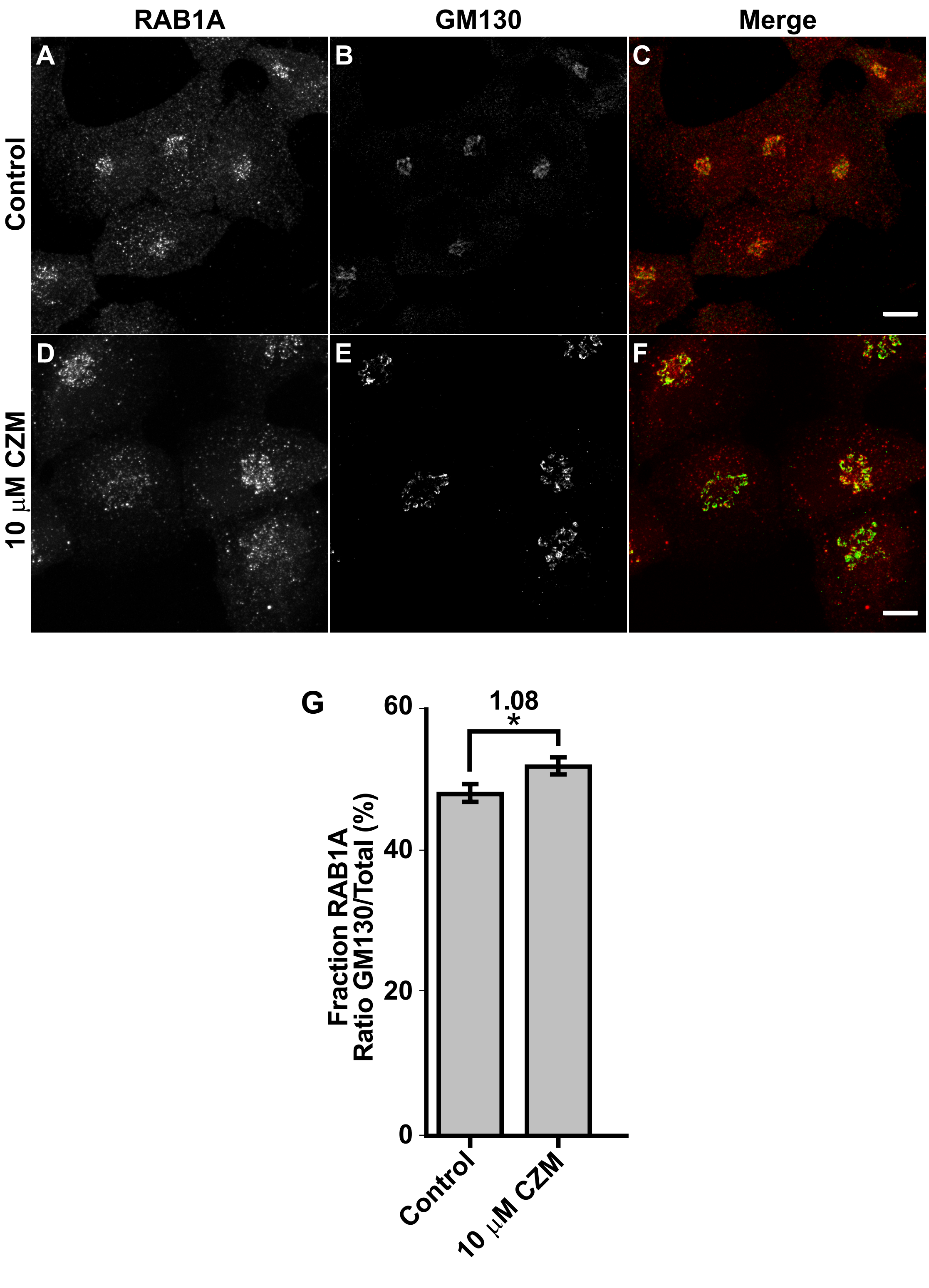
RAB1A
GM130
Merge
A
B
C
D
E
F
Control
10 μM CZM
G
1.08
*
Fraction RAB1A
Ratio GM130/Total (%)
60
40
20
0
Control
10 μM CZM

Figure S7

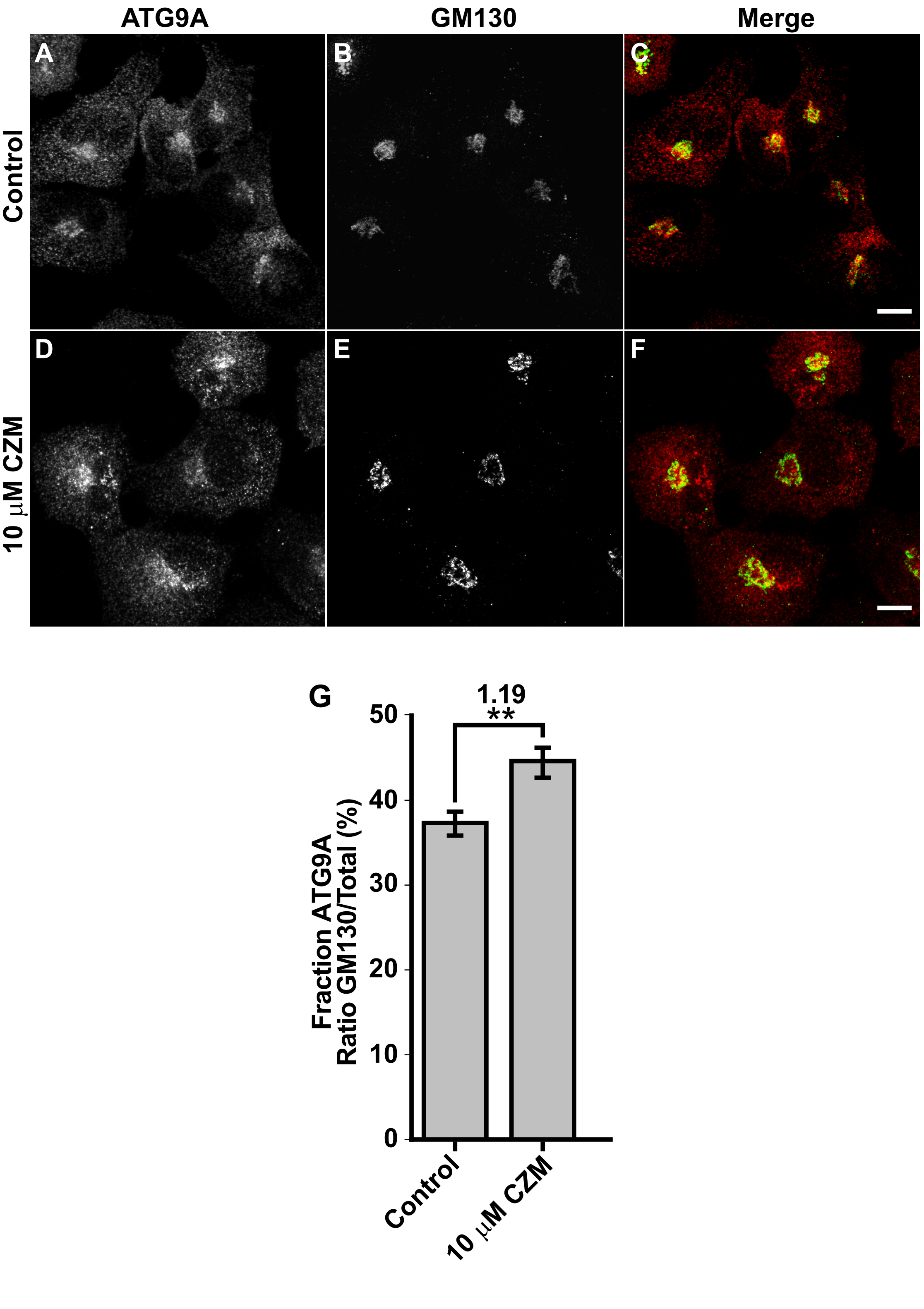

ATG9A
GM130
Merge
A
B
C
D
E
F
Control
10 μM CZM
G
1.19
**
Fraction ATG9A
Ratio GM130/Total (%)
50
40
30
20
10
0
Control
10 μM CZM

Figure S8

Target List High-Content siRNA Screening "Ubiquitinome"

Gene Symbol	NCBI Reference Sequence
**PSMD14**	NM_005805
**PSMD7**	NM_002811
**KCTD3**	NM_016121
**UBE2E2**	NM_152653
**NACA**	NM_005594
**EPN1**	NM_013333
**SIAH2**	NM_005067
**UBA52**	NM_003333
**EIF3S5**	NM_003754
**MARK4**	NM_031417
**UBC**	NM_021009
**UBE2E3**	NM_182678
**RBM6**	NM_005777
**ZBTB12**	NM_181842
**PHF12**	NM_020889
**DNAJB2**	NM_001039550
**BIRC7**	NM_022161
**KBTBD2**	NM_015483
**RYBP**	NM_012234
**NEURL**	NM_004210
**HECTD3**	NM_024602
**ZNF592**	NM_014630
**FLJ25076**	XM_940609
**TNK2**	NM_001010938
**PRPF8**	NM_006445
**TRIM31**	NM_052816
**UCHL3**	NM_006002
**UBE2D4**	NM_015983
**MDM4**	NM_002393
**TRIM63**	NM_032588
**UBE2J1**	NM_016021
**DDI2**	NM_032341
**RNF146**	NM_030963
**CHD5**	NM_015557
**CISH**	NM_145071
**SENP8**	NM_145204
**FLJ43374**	NM_198582
**HIP2**	NM_005339
**UBE2V1**	NM_001032288
**ZNF216**	NM_006007
**KIAA1536**	NM_020898
**WDR11**	NM_018117
**SH3RF2**	NM_152550
**RAI17**	NM_020338
**PCGF2**	NM_007144
**RNF34**	NM_025126
**FBXO4**	NM_012176
**RNF141**	NM_016422
**BRPF3**	NM_015695
**CAND2**	XM_944849
**UBXD4**	NM_181713
**TRIM23**	NM_033228
**FAU**	NM_001997
**RSPRY1**	NM_133368
**TRIM42**	NM_152616
**FBXW10**	NM_031456
**MDM2**	NM_006879
**TOM1**	NM_005488
**SOCS3**	NM_003955
**FBXO44**	NM_183413
**RNF130**	NM_018434
**CREBL1**	NM_004381
**RNF39**	NM_170770
**HRC**	NM_002152
**VPRBP**	NM_014703
**FAF1**	NM_131917
**PSMD4**	NM_002810
**RNF187**	XM_047499
**SHFM3**	NM_022039
**EIF2AK4**	NM_001013703
**SUMO2**	NM_001005849
**FLJ20280**	NM_017741
**TCEB1**	NM_005648
**RAMP**	NM_016448
**PHF23**	NM_024297
**KUA-UEV**	NM_003349
**NICE-4**	NM_014847
**UBB**	NM_018955
**ZBTB16**	NM_001018011
**OTUD5**	NM_017602
**ASPSCR1**	NM_024083
**LOC652557**	XM_942059
**SF3B3**	NM_012426
**LGALS3BP**	NM_005567
**TRIM56**	NM_030961
**SAE1**	NM_005500
**KLHL1**	NM_020866
**MNAT1**	NM_002431
**KCTD17**	NM_024681

**PCF11**	NM_015885
**USP42**	NM_032172
**PHF21A**	NM_016621
**TRIM74**	NM_198853
**KLHL7**	NM_001031710
**RNF32**	NM_030936
**TRIM60**	NM_152620
**UBE2D2**	NM_003339
**UFC1**	NM_016406
**TA-KRP**	NM_032505
**UBE2J2**	NM_194457
**RANBP9**	NM_005493
**BMI1**	NM_005180
**PC326**	NM_001017977
**EIF3S3**	NM_003756
**CDC34**	NM_004359
**UBXD3**	NM_152376
**KCND1**	NM_004979
**PEX10**	NM_002617
**USP52**	NM_014871
**PLAA**	NM_001031689
**BMI1**	NM_005180
**PDZRN4**	NM_013377
**FLJ25555**	NM_152345
**BIRC2**	NM_001166
**STAMBPL1**	NM_020799
**MIB2**	NM_080875
**PIAS1**	NM_016166
**TRIM7**	NM_203294
**LL0XNC01-237H1.1**	NM_001031834
**USP9Y**	NM_004654
**TRIM33**	NM_033020
**LOC646862**	XM_929820
**TSG101**	NM_006292
**TRIM6-TRIM34**	NM_001003819
**FBXO2**	NM_012168
**WDR26**	NM_025160
**SKP1A**	NM_170679
**CUL5**	NM_003478
**C13ORF22**	NM_005800
**TRAF7**	NM_206835
**RBM10**	NM_152856
**FBXL20**	NM_032875
**ASB18**	NM_212556
**LONRF2**	NM_198461

**USP6**	NM_004505
**KCMF1**	NM_020122
**VPS11**	NM_021729
**KRTAP5-9**	NM_005553
**USP26**	NM_031907
**USP16**	NM_001001992
**USP8**	NM_005154
**KCTD12**	NM_138444
**USP10**	NM_005153
**RPS27A**	NM_002954
**UBE2M**	NM_003969
**LOC51035**	NM_015853
**RKHD1**	NM_203304
**HACE1**	NM_020771
**FANCD2**	NM_001018115
**YAF2**	NM_005748
**SIP**	NM_001007214
**RNF44**	NM_014901
**ZNF151**	NM_003443
**ZRANB1**	NM_017580
**TCEB2**	NM_207013
**TCEB3**	NM_003198
**RNF186**	NM_019062
**LOC441061**	XM_941081
**BTBD14A**	NM_144653
**LOC646862**	XM_929820
**LMO7**	NM_005358
**FLJ10719**	NM_018193
**AB026190**	NM_014458
**UBE2NL**	NM_001012989
**FBXO5**	NM_012177
**HECTD1**	NM_015382
**DKFZP547C195**	NM_207343
**TRIM32**	NM_012210
**HECW1**	NM_015052
**AIRE**	NM_000659
**KCNC1**	NM_004976
**UNK**	NM_001080419
**TRIM8**	NM_030912
**SBBI54**	NM_138334
**C20ORF18**	NM_031227
**RNF167**	NM_015528
**KCNA1**	NM_000217
**OTUB2**	NM_023112
**SPSB2**	NM_032641

**GGA3**	NM_014001
**SPSB3**	NM_080861
**SHARPIN**	NM_030974
**HGS**	NM_004712
**TIP120A**	NM_018448
**UEVLD**	NM_018314
**ATG10**	NM_031482
**CHD4**	NM_001273
**FBXO34**	NM_017943
**UBQLN2**	NM_013444
**ZNF185**	NM_007150
**FBXO17**	NM_148169
**ANKFY1**	NM_020740
**USP43**	XM_371015
**PARC**	NM_015089
**HR**	NM_018411
**ZNF297B**	NM_014007
**UBL5**	NM_024292
**PHF19**	NM_001009936
**KCTD5**	NM_018992
**MARCH4**	NM_020814
**ASB14**	NM_130387
**FLJ34154**	NM_173813
**HUWE1**	NM_031407
**ZBTB1**	NM_014950
**FBXL22**	NM_203373
**RNF207**	NM_207396
**RFWD3**	NM_018124
**UBE2L6**	NM_004223
**PHF16**	NM_014735
**MARCH8**	NM_001002266
**FBXL14**	NM_152441
**BACH2**	NM_021813
**DDB1**	NM_001923
**TRIM2**	NM_015271
**RNF38**	NM_194331
**DTX3**	NM_178502
**SH3MD4**	XM_293090
**NSMCE1**	NM_145080
**PML**	NM_033247
**C9ORF60**	NM_006336
**SENP1**	NM_014554
**SIAH1**	NM_003031
**CCL20**	NM_004591
**COPS7A**	NM_016319

**USP45**	XM_371838
**PHF5A**	NM_032758
**UBE4B**	NM_006048
**KLHDC5**	NM_020782
**KCNC3**	NM_004977
**RNF121**	NM_194452
**PARK7**	NM_007262
**UBE2N**	NM_003348
**CUEDC1**	NM_017949
**BARD1**	NM_000465
**TAB3**	NM_198312
**FBXL13**	NM_145032
**MID2**	NM_052817
**FBXW5**	NM_178226
**PRPF19**	NM_014502
**WDR40A**	NM_015397
**RAB40C**	NM_021168
**FBXL12**	NM_017703
**BTBD9**	NM_152733
**PHF6**	NM_032335
**LRRC29**	NM_001004055
**USP54**	NM_152586
**HSHIN6**	NM_207320
**MYO6**	NM_004999
**FBXL2**	NM_012157
**FBXO39**	NM_153230
**UBE2H**	NM_182697
**ZNF131**	NM_003432
**LATS1**	NM_004690
**EEA1**	NM_003566
**ITCH**	NM_031483
**BAZ1B**	NM_032408
**RFP**	NM_030950
**TOM1L1**	NM_005486
**BIRC3**	NM_182962
**MSL2L1**	NM_018133
**BRE**	NM_199191
**WDR71**	NM_025155
**NOSIP**	NM_015953
**RUFY1**	NM_025158
**COPS4**	NM_016129
**LOC392188**	XM_373238
**TRIM29**	NM_058193
**VPS41**	NM_080631
**ATG16L1**	NM_198890

**FEM1C**	NM_020177
**ATG7**	NM_006395
**C17ORF27**	NM_020914
**RNF215**	NM_001017981
**SYVN1**	NM_032431
**TRIM73**	NM_198924
**KBTBD4**	NM_018095
**CPSF1**	NM_013291
**NEURL2**	NM_080749
**FBXO3**	NM_033406
**NLRC5**	NM_032206
**KCTD15**	NM_024076
**HIC2**	NM_015094
**RNF12**	NM_016120
**KIAA0363**	XM_001717181
**SPRYD5**	NM_032681
**CUL4A**	NM_003589
**LOC92312**	XM_937993
**HOZFP**	NM_152995
**PHF11**	NM_016119
**LOC653192**	XM_926437
**REC14**	NM_025234
**TZFP**	NM_014383
**UBE2A**	NM_181762
**RNF165**	NM_152470
**LNX2**	NM_153371
**RNF208**	NM_031297
**FBXW12**	NM_207102
**SMARCA3**	NM_139048
**FEM1B**	NM_015322
**MLL3**	NM_021230
**RHOBTB2**	NM_015178
**SFRS2IP**	NM_004719
**USP3**	NM_006537
**DPF1**	NM_004647
**NEDD4**	NM_006154
**PHF13**	NM_153812
**REV3L**	NM_002912
**NEDD4L**	NM_015277
**M96**	NM_007358
**GGA2**	NM_015044
**SUMO3**	NM_006936
**ASB6**	NM_177999
**TRIM36**	NM_018700
**PIAS4**	NM_015897

**MGC3123**	NM_024107
**FBXO11**	NM_012167
**TRAF5**	NM_001033910
**BRIP1**	NM_032043
**TNIP2**	NM_024309
**RANBP2**	NM_006267
**ASB12**	NM_130388
**ANUBL1**	NM_001128324
**KLHL6**	NM_130446
**UBE2F**	NM_080678
**NPL4**	NM_017921
**RBAF600**	NM_020765
**SNF1LK**	NM_173354
**FBXL16**	NM_153350
**SUMO4**	NM_001002255
**KCNA4**	NM_002233
**TIF1**	NM_003852
**USP39**	NM_006590
**SOCS4**	NM_080867
**USP27X**	XM_372213
**USP7**	NM_003470
**FBXL17**	NM_022824
**DKFZP564O0463**	NM_015420
**ARIH1**	NM_005744
**USP41**	XM_036729
**TNFAIP3**	NM_006290
**FBXO43**	NM_001029860
**ZNF179**	NM_007148
**PCGF1**	NM_032673
**ZNF265**	NM_203350
**KCNA3**	NM_002232
**TRIM25**	NM_005082
**CDC20**	NM_001255
**DCUN1D1**	NM_020640
**ZBTB2**	NM_020861
**PDZRN3**	NM_015009
**LZTR1**	NM_006767
**GCL**	NM_178439
**CUL1**	NM_003592
**PHF17**	NM_199320
**KCTD10**	NM_031954
**RWDD3**	NM_001128142
**USP15**	NM_006313
**PHF7**	NM_173341
**MLLT10**	NM_001009569

**TRIM9**	NM_052978
**MARCH7**	NM_022826
**RNF43**	NM_017763
**USP22**	XM_042698
**MARCH6**	NM_005885
**UBE2Q1**	NM_017582
**PCGF3**	NM_006315
**SYTL3**	NM_001009991
**RNF148**	NM_198085
**UBADC1**	NM_016172
**STAMBP**	NM_201647
**UBE1L**	NM_003335
**TRIM46**	NM_025058
**DDB2**	NM_000107
**UBE2L3**	NM_003347
**TBL1XR1**	NM_024665
**CUL2**	NM_003591
**WDSUB1**	NM_152528
**BMSC-UBP**	NM_201265
**PPIL2**	NM_014337
**FLJ13456**	NM_024646
**BTBD12**	NM_032444
**KCTD13**	NM_178863
**SKP2**	NM_032637
**KCNS3**	NM_002252
**UBE1**	NM_153280
**UBE3A**	NM_130838
**LOC196394**	NM_207337
**HERC1**	NM_003922
**RNF6**	NM_183045
**ATG3**	NM_022488
**IMPACT**	NM_018439
**UBE3B**	NM_183415
**KIAA0459**	XM_375697
**CNOT4**	NM_001008225
**TRIM40**	NM_138700
**SIK2**	NM_015191
**BRPF1**	NM_004634
**ZFPL1**	NM_006782
**ZNF313**	NM_018683
**AOF1**	NM_153042
**TRIM71**	NM_001039111
**TRAF6**	NM_004620
**KLHL13**	NM_033495
**H326**	NM_015726

**HERC2**	NM_004667
**UBE2D1**	NM_003338
**ZBTB39**	NM_014830
**RNF133**	NM_139175
**USP21**	NM_012475
**UBE3C**	NM_014671
**NIPBL**	NM_015384
**FBXO38**	NM_030793
**FLJ11280**	NM_001040217
**ASB11**	NM_001012428
**FLJ40411**	NM_001080504
**LOC342897**	NM_001001414
**UBE2I**	NM_194260
**CXXC1**	NM_014593
**UBE1C**	NM_198197
**WSB2**	NM_018639
**SMURF1**	NM_181349
**NXF2**	NM_022053
**FLJ46299**	NM_207335
**FBXO27**	NM_178820
**WDR21**	NM_181341
**D8S2298E**	NM_005671
**TRIP**	NM_005879
**RNF8**	NM_183078
**PCGF6**	NM_032154
**TRIM58**	NM_015431
**JARID1C**	NM_004187
**PHF10**	NM_018288
**ZNF499**	NM_032792
**NEDD8**	NM_006156
**DCUN1D5**	NM_032299
**TRIAD3**	NM_019011
**PJA1**	NM_001032396
**JARID1D**	NM_004653
**UBE2C**	NM_181803
**HECW2**	NM_020760
**KCNA7**	NM_031886
**UBE1DC1**	NM_024818
**PHF20**	NM_016436
**KEAP1**	NM_012289
**LOC441920**	XM_497731
**RWDD2**	NM_033411
**RNF157**	NM_052916
**KIAA0795**	NM_025010
**FBXO31**	NM_024735

**C20ORF11**	NM_017896
**PHF1**	NM_002636
**KIAA0644**	NM_014817
**TRIM45**	NM_025188
**ZNF547**	NM_173631
**LOC342931**	XM_292796
**ZNF294**	NM_015565
**UBE2G1**	NM_182682
**KBTBD11**	NM_014867
**RNF31**	NM_017999
**RNF169**	XM_495886
**MGC20470**	NM_145053
**CDC27**	NM_001256
**TRAF4**	NM_145751
**KCTD7**	NM_153033
**TULP4**	NM_001007466
**PIAS2**	NM_173206
**IPP**	NM_005897
**ASB10**	NM_080871
**UHRF2**	NM_152896
**SENP7**	NM_020654
**MOCS3**	NM_014484
**UBE2B**	NM_003337
**USP14**	NM_001037334
**ZNF645**	NM_152577
**LOC283219**	NM_001029859
**UBE2O**	NM_022066
**UBE2R2**	NM_017811
**DPF3**	NM_012074
**LOC648245**	XM_942858
**LOC648245**	XM_942858
**RAG1**	NM_000448
**C10ORF3**	NM_018131
**STUB1**	NM_005861
**STAM**	NM_003473
**SOCS1**	NM_003745
**IRF2BP1**	NM_015649
**KIAA0999**	NM_025164
**PHF3**	NM_015153
**COPS8**	NM_006710
**UBAP1**	NM_016525
**KCTD6**	NM_001128214
**RAD23A**	NM_005053
**DMRT3**	NM_021240
**KBTBD9**	XM_496546

**BIRC6**	NM_016252
**IBRDC2**	NM_182757
**MARK1**	NM_018650
**TANK**	NM_133484
**LOC196394**	NM_207337
**POLI**	NM_007195
**JARID1B**	NM_006618
**NDP52**	NM_005831
**SOCS7**	NM_014598
**KBTBD1**	NM_001003760
**KCND2**	NM_012281
**SENP3**	NM_015670
**DRE1**	NM_017644
**LONRF1**	NM_152271
**USP29**	NM_020903
**MKLN1**	NM_013255
**FLJ35834**	NM_178827
**DCUN1D2**	NM_001014283
**FBXO47**	NM_001008777
**CYLD**	NM_015247
**KLHL21**	NM_014851
**CXORF53**	NM_001018055
**SOCS6**	NM_004232
**RNF41**	NM_194359
**HAN11**	NM_001003725
**BAZ2B**	NM_013450
**RNF126**	NM_017876
**DKFZP761G2113**	NM_001098833
**WDR24**	NM_032259
**MID1**	NM_033290
**UBPH**	NM_019116
**LOC643904**	XM_927169
**LOC644006**	XM_929433
**KCNA2**	NM_004974
**BRODL**	NM_153252
**C14ORF4**	NM_024496
**ADRM1**	NM_175573
**TDRD3**	NM_030794
**KCNV1**	NM_014379
**KCNC2**	NM_139137
**KLHL9**	NM_018847
**WWP1**	NM_007013
**PIAS3**	NM_006099
**UBD**	NM_006398
**USP13**	NM_003940

**FBXL7**	NM_012304
**ZNF336**	NM_022482
**ZFAND6**	NM_019006
**RCBTB1**	NM_018191
**UBXD1**	NM_025241
**IBRDC1**	NM_152553
**FLJ11078**	NM_018316
**UBE2S**	NM_014501
**LOC130617**	NM_138802
**WHSC1**	NM_007331
**PPIL5**	NM_203467
**DZIP3**	NM_014648
**DCUN1D3**	NM_173475
**HIC1**	NM_006497
**MED8**	NM_001001654
**ZNF482**	NM_006626
**PHF21B**	NM_138415
**TRIM15**	NM_052812
**KLHL15**	NM_030624
**ZNRF1**	NM_032268
**LOC645402**	XM_928448
**FLJ31951**	NM_144726
**KCTD14**	NM_023930
**SYTL4**	NM_080737
**ASC1P100**	NM_032204
**LINCR**	XM_930227
**C16orf28**	NM_023076
**FBXO46**	XM_371179
**TRIM75**	XM_939332
**AUP1**	NM_181575
**RNF40**	NM_014771
**FBXO18**	NM_178150
**CCNB1IP1**	NM_182849
**TOLLIP**	NM_019009
**KIAA1542**	NM_020901
**CHC1L**	NM_001268
**FBXL11**	NM_012308
**TRIM37**	NM_001005207
**ZBTB33**	NM_006777
**OTUD6B**	NM_016023
**RNF183**	NM_145051
**UBTD1**	NM_024954
**ASB7**	NM_024708
**SENP2**	NM_021627
**HERC3**	NM_014606

**KBTBD5**	NM_152393
**TNIP1**	NM_006058
**LOC642219**	XM_936370
**MGC5306**	NM_024116
**ZNF295**	NM_020727
**HKR3**	NM_005341
**RNF182**	NM_152737
**HERPUD1**	NM_001010990
**POLK**	NM_016218
**TRIM3**	NM_033278
**LOC137886**	NM_001077619
**VPS18**	NM_020857
**SNRK**	NM_017719
**EPS15**	NM_001981
**RNF2**	NM_007212
**ZNF278**	NM_032051
**DTX3L**	NM_138287
**DAXX**	NM_001350
**LOC388419**	NM_001080466
**PRICKLE1**	NM_153026
**LOC342931**	XM_292796
**RFPL1**	NM_021026
**FBXL19**	NM_019085
**DDI1**	NM_001001711
**FBXO9**	NM_012347
**TRIM62**	NM_018207
**MYLIP**	NM_013262
**HECTD2**	NM_173497
**TRIM26**	NM_003449
**FBXL10**	NM_001005366
**PCGF5**	NM_032373
**BFAR**	NM_016561
**ZNF330**	NM_014487
**SOC**	NM_001077262
**ZBTB3**	NM_024784
**UBQLN1**	NM_053067
**WHSC1L1**	NM_017778
**LGR6**	NM_001017404
**USP5**	NM_003481
**ASB5**	NM_080874
**DERL1**	NM_024295
**DPF2**	NM_006268
**MLL2**	NM_003482
**FBXO42**	NM_018994
**SENP5**	NM_152699

**TRIM72**	NM_001008274
**C10ORF46**	NM_153810
**KCNC4**	NM_153763
**MLLT6**	NM_005937
**TRIM38**	NM_006355
**TRIM55**	NM_184087
**SOLH**	NM_005632
**USP25**	NM_013396
**MAP3K7IP2**	NM_015093
**RNF17**	NM_031994
**BTBD3**	NM_181443
**SSA1**	NM_003141
**ZBTB9**	NM_152735
**TRIM43**	NM_138800
**SCA7**	NM_000333
**BTBD1**	NM_001011885
**TRIM49**	NM_020358
**KIAA1811**	NM_032430
**BTBD2**	NM_017797
**ASB3**	NM_145863
**C6ORF113**	NM_145062
**FBXO25**	NM_012173
**TRIM54**	NM_187841
**ZFP161**	NM_003409
**PHGDHL1**	NM_177967
**FBXL6**	NM_024555
**UBE1L2**	NM_018227
**UBE2V2**	NM_003350
**EDD1**	NM_015902
**LOC51255**	NM_016494
**USP11**	NM_004651
**C14ORF130**	NM_175748
**CDC23**	NM_004661
**MGC10765**	NM_024345
**LOC652673**	XM_942254
**KIAA0804**	NM_015303
**LOC153918**	NM_001013623
**LOC164153**	NM_203412
**VHL**	NM_198156
**FLJ32642**	NM_152415
**RFP2**	NM_005798
**AKTIP**	NM_022476
**USP44**	NM_032147
**JOSD1**	NM_014876
**COPS7B**	NM_022730

**FLJ10916**	NM_018271
**SENP6**	NM_015571
**KIAA1333**	NM_017769
**BTBD14B**	NM_052876
**LMTK3**	XM_055866
**KIAA1164**	NM_001040453
**TTC3**	NM_001001894
**USP20**	NM_001008563
**RNF13**	NM_183384
**TOM1L2**	NM_144678
**MARCH1**	NM_017923
**UBE2G2**	NM_003343
**RNF10**	NM_014868
**LNX1**	NM_032622
**UBE2U**	NM_152489
**PXMP3**	NM_000318
**UBE2T**	NM_014176
**SUMO1**	NM_001005781
**SCEL**	NM_003843
**KCTD16**	NM_020768
**CUL3**	NM_003590
**RAB40A**	NM_080879
**ZNF509**	NM_145291
**CDC16**	NM_003903
**KLHL5**	NM_199039
**LOC339745**	NM_001001664
**TRIM67**	NM_001004342
**WDR59**	NM_030581
**ASB13**	NM_024701
**NSD1**	NM_172349
**BTBD5**	NM_017658
**RCHY1**	NM_001009922
**MARCH2**	NM_001005416
**NUP153**	NM_005124
**GTF2H2**	NM_001515
**RC3H2**	NM_018835
**LOC345930**	XM_941136
**USP48**	NM_001032730
**USP35**	XM_290527
**FBXO15**	NM_152676
**USP9X**	NM_021906
**RNF111**	NM_017610
**CBX4**	NM_003655
**ZNF598**	NM_178167
**CBLL1**	NM_024814

**N4BP2**	NM_018177
**WRNIP1**	NM_130395
**KCNA6**	NM_002235
**BCL6**	NM_001706
**BTBD7**	NM_018167
**UBE2Q2**	NM_173469
**KCNA5**	NM_002234
**USP12**	NM_182488
**EPN2**	NM_148921
**C22ORF3**	NM_012265
**BTBD4**	NM_025224
**CBL**	NM_005188
**UBXD2**	NM_014607
**C1orf166**	NM_024544
**UBAP2**	NM_020867
**HYPK**	NM_016400
**DMRTA1**	NM_022160
**ARIH1**	NM_005744
**CCDC50**	NM_174908
**PARP11**	NM_020367
**CCNF**	NM_001761
**COPS6**	NM_006833
**USP18**	NM_017414
**RNF168**	NM_152617
**BTRC**	NM_003939
**LMO6**	NM_006150
**PHF14**	NM_001007157
**OTUD1**	XM_166659
**KCNG1**	NM_002237
**KCTD9**	NM_017634
**PROSAPIP2**	NM_014726
**ZFYVE20**	NM_022340
**TNFRSF25**	NM_148970
**TRIM28**	NM_005762
**RKHD3**	NM_032246
**ASB2**	NM_016150
**ATF6**	NM_007348
**MYSM1**	XM_055481
**ETEA**	NM_014613
**USP2**	NM_171997
**SHPRH**	NM_173082
**PHF20L1**	NM_032205
**UHRF1**	NM_013282
**PRICKLE2**	NM_198859
**LOC120126**	XM_936270

**EGLN2**	NM_080732
**HERC4**	NM_001017972
**USP32**	NM_032582
**MGC29814**	NM_182565
**OTUD7**	NM_130901
**C6ORF49**	NM_013397
**ZSWIM2**	NM_182521
**UBL4**	NM_014235
**USP28**	NM_020886
**ZNRF2**	NM_147128
**ANKRD13**	NM_033121
**ABTB2**	NM_145804
**TRAF3**	NM_003300
**STK29**	NM_003957
**KLHL12**	NM_021633
**MARCH3**	NM_178450
**MGC10198**	NM_152682
**ARIH2**	NM_006321
**C7ORF21**	NM_031434
**ATG5**	NM_004849
**EPS15L1**	NM_021235
**KLHL8**	NM_020803
**FSD1L**	NM_031919
**FBXW11**	NM_033645
**ASB9**	NM_001031739
**FBXW9**	NM_032301
**KLHL11**	NM_018143
**MARCH9**	NM_138396
**RNF14**	NM_183399
**RFPL3**	NM_006604
**LATS2**	NM_014572
**LOC340359**	NM_001081675
**TRIM39**	NM_172016
**INTS12**	NM_020395
**LOC120126**	XM_936270
**ERCC5**	NM_000123
**YOD1**	NM_018566
**USP49**	NM_018561
**NHLRC1**	NM_198586
**RFC1**	NM_002913
**MARK2**	NM_004954
**FBXO28**	NM_015176
**TRIM64**	XM_061890
**SQSTM1**	NM_003900
**WSB1**	NM_134265

**USP30**	NM_032663
**FBXO21**	NM_015002
**BTBD8**	NM_183242
**UBQLN3**	NM_017481
**VCP**	NM_007126
**MIB1**	NM_020774
**LOC399940**	XM_374920
**RNF190**	NM_152598
**USP1**	NM_001017416
**MEFV**	NM_000243
**TRIM11**	NM_145214
**FBXL4**	NM_012160
**BAP1**	NM_004656
**TRIM17**	NM_001024941
**UBA2**	NM_005499
**TTRAP**	NM_016614
**EPN3**	NM_017957
**USP50**	NM_203494
**ANAPC10**	NM_014885
**FBXL15**	NM_024326
**KLHL14**	NM_020805
**FRBZ1**	NM_194314
**TRIM68**	NM_018073
**DUB3**	NM_201402
**TNFAIP1**	NM_021137
**BAHD1**	NM_014952
**ZNRF3**	XM_290972
**SOCS5**	NM_014011
**FLJ14981**	NM_032868
**TRIM5**	NM_033092
**UFD1L**	NM_001035247
**KBTBD6**	NM_152903
**RNF125**	NM_017831
**KBTBD3**	NM_152433
**MKRN3**	NM_005664
**KCNA10**	NM_005549
**ATRX**	NM_138270
**DKFZP547N043**	NM_032018
**XPA**	NM_000380
**USP38**	NM_032557
**BRCA1**	NM_007298
**UBASH3A**	NM_001001895
**RNF113A**	NM_006978
**ZFP67**	NM_015872
**ENC1**	NM_003633

**HBXAP**	NM_016578
**KIAA1018**	NM_014967
**UBR1**	NM_174916
**LOC51136**	NM_016125
**KCNRG**	NM_173605
**KLHDC2**	NM_014315
**MGC33190**	NM_152749
**TRIM59**	NM_173084
**MYNN**	NM_018657
**FBXL3A**	NM_012158
**ANAPC2**	NM_013366
**MKRN2**	NM_014160
**RNF122**	NM_024787
**NEURL1B**	NM_001142651
**KIAA1718**	NM_030647
**PHF15**	NM_015288
**KLHL3**	NM_017415
**OPTN**	NM_021980
**ZNF46**	NM_006977
**RAP80**	NM_016290
**ATG12**	NM_004707
**C6ORF157**	NM_198920
**RBX1**	NM_014248
**ANKRD9**	NM_152326
**IVNS1ABP**	NM_006469
**RNF138**	NM_198128
**POLH**	NM_006502
**DMRTA2**	NM_032110
**FBXL18**	NM_024963
**UCHL5**	NM_015984
**FBXO30**	NM_032145
**RNF149**	NM_173647
**HERC5**	NM_016323
**OTUD4**	NM_017493
**USP31**	NM_020718
**ZA20D1**	NM_020205
**OTUB1**	NM_017670
**FBXW8**	NM_012174
**NYREN18**	NM_016118
**MGC46534**	NM_153340
**KCTD4**	NM_198404
**RNF135**	NM_197939
**ZNF364**	NM_014455
**CDC26**	NM_139286
**MAP3K1**	XM_042066

**USP53**	NM_019050
**MKRN1**	NM_013446
**HDAC6**	NM_006044
**BACH1**	NM_001011545
**ASB16**	NM_080863
**FLN29**	NM_006700
**CUL7**	NM_014780
**MGC71999**	NM_199290
**DTX1**	NM_004416
**FBXO36**	NM_174899
**BIRC8**	NM_033341
**DHX57**	NM_198963
**RC3H1**	NM_172071
**RFWD2**	NM_001001740
**BTBD11**	NM_152322
**KCTD8**	NM_198353
**TRIM35**	NM_015066
**RAB40B**	NM_006822
**ZRANB3**	NM_032143
**USP19**	NM_006677
**FBXL8**	NM_018378
**RHOBTB3**	NM_014899
**USP47**	NM_017944
**BTBD6**	NM_033271
**SMARCAD1**	NM_020159
**APM-1**	XM_113971
**NEIL3**	NM_018248
**KIAA0478**	NM_014870
**RING1**	NM_002931
**VCPIP1**	NM_025054
**CGRRF1**	NM_006568
**MGC22679**	NM_144711
**RFFL**	NM_001017368
**DCUN1D4**	NM_015115
**ANAPC1**	NM_022662
**GMRP-1**	NM_032320
**RABGEF1**	NM_014504
**LOC645836**	XM_001720764
**FLJ34960**	NM_153270
**USP17**	NM_001105662
**FLJ31031**	NM_182533
**VPS13D**	NM_018156
**KIAA1900**	NM_052904
**ANKRD9**	NM_152326
**UBL3**	NM_007106

**UBE2W**	NM_001001482
**FBXO32**	NM_148177
**G1P2**	NM_005101
**KIAA1582**	NM_018996
**DTX2**	NM_020892
**ZBTB26**	NM_020924
**USP34**	NM_014709
**TOPORS**	NM_005802
**RNF150**	NM_020724
**LOC90637**	NM_182491
**C1ORF6**	NM_020131
**KLHDC3**	NM_057161
**FLJ13063**	NM_001105247
**FLJ39827**	NM_152424
**LOC731049**	XM_001724228
**FBXO8**	NM_012180
**MARCH5**	NM_017824
**KCTD1**	NM_198991
**RNF139**	NM_007218
**ASB8**	NM_024095
**ANKIB1**	XM_377955
**PDC**	NM_022576
**RNF128**	NM_024539
**TBL1XR1**	NM_024665
**LOC285498**	NM_194439
**TRIM47**	NM_033452
**ASB15**	NM_080928
**USP46**	NM_022832
**MGRN1**	NM_015246
**SH3MD2**	NM_020870
**IBRDC3**	NM_153341
**RAD18**	NM_020165
**TRIM52**	NM_032765
**ZBTB11**	NM_014415
**C16ORF44**	NM_024731
**RNF175**	NM_173662
**MGC10067**	NM_145049
**TRIM34**	NM_001003827
**SPSB1**	NM_025106
**KIAA0317**	NM_014821
**PEX12**	NM_000286
**TRFP**	NM_004275
**MGC2629**	NM_032522
**RNF103**	NM_005667
**FLJ13096**	NM_025000

**UBE2E1**	NM_182666
**ZNF297**	NM_005453
**KLHL4**	NM_057162
**DTX4**	XM_166213
**RNF144**	NM_014746
**LOC100134427**	XM_001719136
**WWP2**	NM_199424
**WDR23**	NM_181357
**TAX1BP1**	NM_006024
**LOC123103**	NM_001109997
**FBXO10**	XM_291314
**OIT3**	NM_152635
**TBL1X**	NM_005647
**UBR2**	NM_015255
**OSTM1**	NM_014028
**CGI-62**	NM_016010
**MARK3**	NM_002376
**RWDD1**	NM_015952
**LOC554251**	NM_001024680
**CBLC**	NM_012116
**USP33**	NM_201626
**IBTK**	NM_015525
**HSPC056**	NM_014154
**RFPL2**	NM_006605
**GPS1**	NM_004127
**NSFL1C**	NM_182483
**TEX27**	NM_021943
**FBXO24**	NM_012172
**LOC652591**	XM_942113
**RNF151**	XM_370927
**E4F1**	NM_004424
**RNF170**	NM_030954
**RNF123**	NM_022064
**RNF185**	NM_152267
**LOC390231**	XM_936301
**ZBTB4**	NM_020899
**KLHL10**	NM_152467
**C1orf164**	NM_018150
**RNF20**	NM_019592
**STAM2**	NM_005843
**WDR22**	NM_003861
**SPSB4**	NM_080862
**ANAPC11**	NM_001002245
**FBXL3P**	NM_012159
**MLL4**	NM_014727

**FBXW7**	NM_001013415
**FLJ14627**	NM_032814
**USP37**	NM_020935
**SOCS2**	NM_003877
**LOC283116**	XM_001720988
**HERC6**	NM_001013000
**KIAA0794**	NM_015562
**FBXO33**	NM_203301
**FBXO16**	NM_172366
**ZBTB24**	NM_014797
**TRIM6**	NM_058166
**C13ORF7**	NM_024546
**IKBKG**	NM_003639
**FANCL**	NM_018062
**USP24**	XM_371254
**KCTD2**	NM_015353
**TRIP12**	NM_004238
**COPS5**	NM_006837
**CHFR**	NM_018223
**ANAPC4**	NM_013367
**TRIML1**	NM_178556
**RNF25**	NM_022453
**UCHL1**	NM_004181
**REV1L**	NM_001037872
**UBE2D3**	NM_181893
**SPOP**	NM_001007228
**MUF1**	NM_006369
**KCTD20**	NM_173562
**SHKBP1**	NM_138392
**UFM1**	NM_016617
**PARK2**	NM_013988
**TRIP15**	NM_004236
**CUL4B**	NM_003588
**KCNG3**	NM_172344
**TRIM65**	NM_173547
**DCST1**	NM_152494
**RNF26**	NM_032015
**RHOBTB1**	NM_001032380
**LOC338692**	NM_207354
**TNIP3**	NM_024873
**USP4**	NM_199443
**ASB1**	NM_016114
**ABTB1**	NM_172028
**UBE2Z**	NM_023079
**TRIM41**	NM_201627

**PHF8**	NM_015107
**ANAPC13**	NM_015391
**TRAF2**	NM_021138
**CBLB**	NM_170662
**TRIM10**	NM_006778
**TRIM69**	NM_080745
**FBXL5**	NM_012161
**LOC643596**	XM_926900
**ZNF650**	NM_172070
**ANAPC5**	NM_016237
**GGA1**	NM_001001561
**RNF152**	NM_173557
**PJA2**	NM_014819
**KIAA1959**	NM_032873
**KCND3**	NM_172198
**BRAP**	NM_006768
**FBXW2**	NM_012164
**ZBTB7**	NM_015898
**FBXO6**	NM_018438
**LOC643596**	XM_926900
**KBTBD7**	NM_032138
**CCIN**	NM_005893
**LOC200933**	XM_946180
**LOC653121**	NM_001040441
**DKFZP761I2123**	NM_174929
**DUB1A**	XM_377830
**TRAF2**	NM_021138
**AMFR**	NM_138958
**RNF166**	NM_178841
**FLJ00012**	NM_033388
**BCL6B**	NM_181844
**TRIM4**	NM_033091
**ASB17**	NM_080868
**MYCBP2**	NM_015057
**ZMYND11**	NM_212479
**KCNB1**	NM_004975
**RNF7**	NM_183237
**NFX1**	NM_147133
**RKHD2**	NM_016626
**C9ORF74**	NM_030914
**USP36**	NM_025090
**C21ORF107**	NM_001007246
**LOC124402**	NM_145253
**DC-UBP**	NM_152277
**UBE4A**	NM_004788

**RNF4**	NM_002938
**TRIM61**	NM_001012414
**LOC646463**	XM_929387
**ISL1**	NM_002202
**LMX1B**	NM_002316
**C21ORF6**	NM_016940
**KLHL22**	NM_032775
**RNF180**	NM_178532
**SMURF2**	NM_022739
**UNKL**	NM_001037125
**RNF5P1**	XM_209913
**FBXO7**	NM_001033024
**LMX1B**	NM_002316
**FEM1A**	NM_018708
**RNF11**	NM_014372
**LONRF3**	NM_024778
**RAPSN**	NM_032645
**RFPL4B**	NM_001013734
**RNF5**	NM_006913
**PHF2**	NM_024517
**FLJ32440**	NM_173685
**LRSAM1**	NM_001005374
**TRIM14**	NM_033219
**ZNF238**	NM_205768
**LOC339451**	NM_198317
**ZBTB38**	XM_172341
**HSPC063**	NM_014155
**USP40**	NM_018218
**ASB4**	NM_016116
**BIRC4**	NM_001167
**LOC642446**	XM_001732877
**UBOX5**	NM_199415
**MJD**	NM_030660
**KCNS1**	NM_002251
**KCNB2**	NM_004770
**DET1**	NM_017996
**RNF24**	NM_007219
**ZNF288**	NM_015642
**FBXO41**	XM_377742
**RNF19**	NM_015435
**APPBP1**	NM_001018160
**KBTBD10**	NM_006063
**COPS3**	NM_003653
**LPXN**	NM_004811
**FLJ12587**	NM_022480

**USP51**	NM_201286
**ZBTB5**	NM_014872
**FBXO40**	NM_016298
**ZBTB10**	NM_023929
**KCNS2**	NM_020697
**RNF113B**	NM_178861
**RBBP6**	NM_032626
**RAD23B**	NM_002874
**TRIM50A**	NM_178125
**MEP50**	NM_024102
**GAN**	NM_022041
**FBXO22**	NM_012170
**TRIM48**	NM_024114
**NXF1**	NM_006362
**TRIM22**	NM_006074
**ZNRF4**	NM_181710
**M17S2**	NM_005899
**NBR1**	NM_031862.4
